# Oldest co-occurrence of *Varanus* and *Python* from Africa—first record of squamates from the early Miocene of Moghra Formation, Western Desert, Egypt

**DOI:** 10.7717/peerj.9092

**Published:** 2020-05-22

**Authors:** Georgios L. Georgalis, Mohamed K. Abdel Gawad, Safiya M. Hassan, Ahmed N. El-Barkooky, Mohamed A. Hamdan

**Affiliations:** 1 Department of Earth Sciences, University of Turin, Turin, Italy; 2 Geology Department, Faculty of Science, Cairo University, Giza, Egypt; 3 Geology Department, Faculty of Science, Beni-Suef University, Beni-Suef, Egypt

**Keywords:** Lizards, Snakes, Neogene, Biogeography, Sympatry, Africa

## Abstract

Lizard and snake remains from the early Miocene (Burdigalian) of the Moghra Formation, Egypt, are described herein. This material comprises the first fossil remains of squamates recovered from the otherwise rich and well known vertebrate assemblage of Moghra. The material pertains to two different genera, the varanid lizard *Varanus* and the pythonid snake *Python* and adds to the so far rather poorly known squamate fossil record from Africa. On the basis of the new remains, Moghra marks the oldest so far described co-occurrence of *Varanus* and *Python* in the African continent. The close sympatry of these two genera in the African fossil record is thoroughly analyzed and discussed, a co-existence, which is still widespread in the extant herpetofauna of the continent. Situated rather close to the so called “Levantine Corridor” and dated at the Burdigalian, practically when Afro-Arabia collided with Eurasia, the Moghra squamate assemblage offers the potential of important insights in the biogeography and dispersal events of vertebrate groups during the early Miocene.

## Introduction

The genera *Varanus* and *Python* are among the most iconic squamates. They are both almost immediately recognizable even to the general public, commonly known as monitor lizards and pythons respectively. *Varanus* comprises the largest extant species of lizards, while certain species of *Python* rank among the longest and heaviest species of snakes (Murphy & Henderson, 1997; Pianka, King & King, 2004). Both *Varanus* and *Python* form important ecological elements to the environments they reside in; there are also significant trophic interactions among the two genera, with documented cases of *Varanus* preying upon *Python* and vice versa (e.g.,  Mash, 1944; Murphy & Henderson, 1997; Chippaux & Jackson, 2019). In sub-Saharan Africa, *Varanus* and *Python* are widespread faunal elements, co-existing together in multiple different environments and biomes, ranging from open savannah to dense tropical rainforest (Pianka, King & King, 2004; O’Shea, 2007). Their fossil record on the African continent is, however, rather scarce, being in fact confined to rather few documented occurrences across the Neogene and Quaternary of the continent (Rage, 1976; Rage, 2003; Rage, 2008; Bailon & Rage, 1994; Clos, 1995; Delfino et al., 2004; Delfino et al., 2018; Head & Müller, 2020). The rarity and inadequate knowledge of their fossil record is readily highlighted by the fact that although multiple species of both genera are present in the extant African herpetofauna (Pianka, King & King, 2004; Wallach, Williams & Boundy, 2014), only two extinct species have been named from the continent, one from each genus, i.e., *Varanus rusingensis* Clos, 1995, from the early Miocene of Kenya and *Python maurus* Rage, 1976, from the middle Miocene of Morocco (Rage, 1976; Clos, 1995).

The current paper describes new remains attributable to *Varanus* and *Python* from the early Miocene (Burdigalian) of the Moghra Formation, Egypt. These are the first squamates described from this locality, which is otherwise well known for its fossil mammals and has proven pivotal for our understanding of vertebrate biogeography and diversity in the African Miocene (Andrews, 1899; Andrews, 1900; Fourtau, 1920; Rasmussen, Tilden & Simons, 1989; Miller & Simons, 1998; Miller, 1999; Sanders & Miller, 2002; Miller et al., 2009; Miller et al., 2014; Cook et al., 2010; Pickford, Miller & El-Barkooky, 2010; Smith, 2013; Morlo et al., 2019). The co-existence of monitor lizards and pythons across the fossil record of Africa is thoroughly discussed. In addition, the global early and middle Miocene distribution of *Varanus* and *Python* is presented on the basis of all so far described occurrences of that age for both genera.

### Geological settings

**Figure 1:**
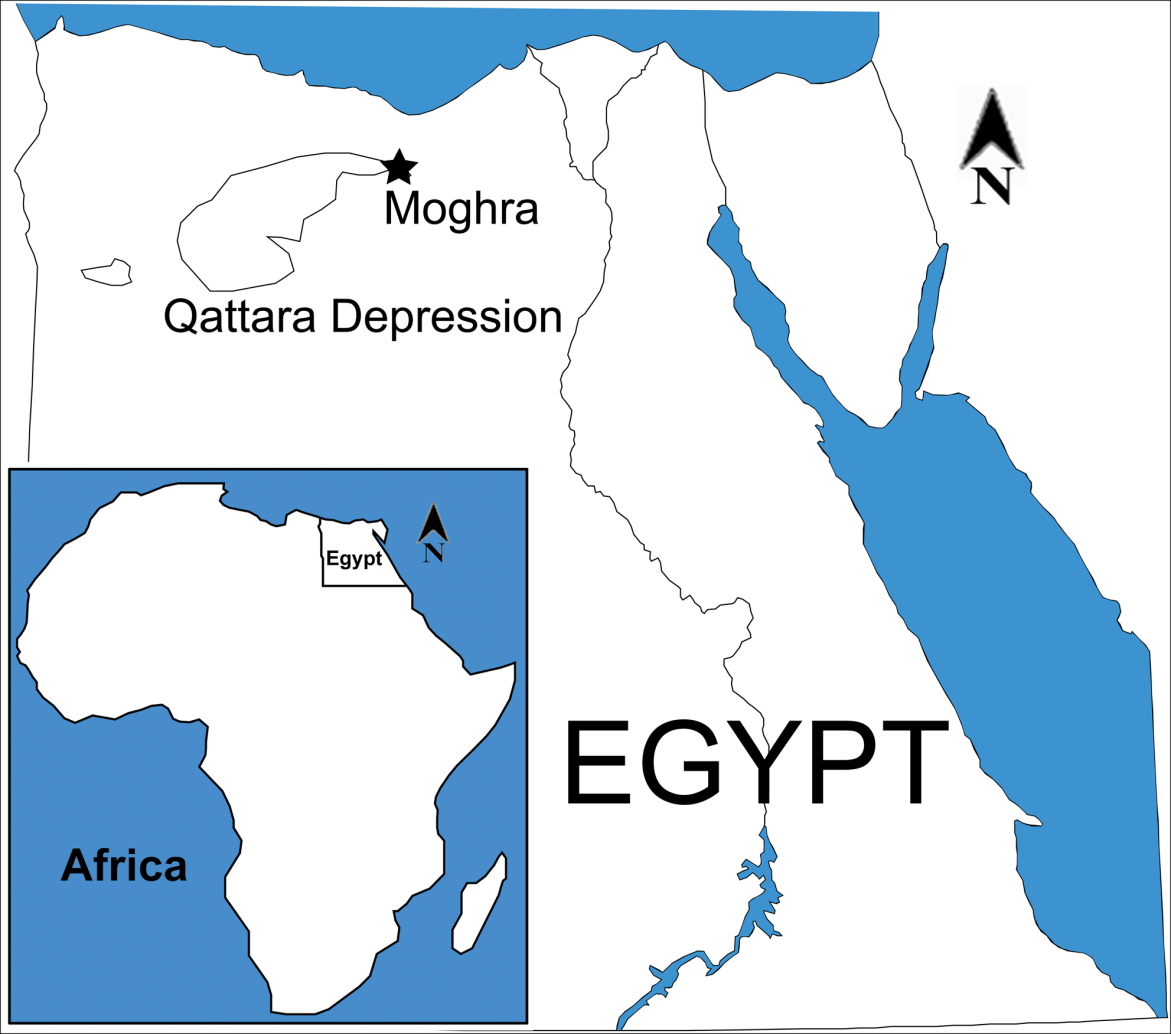
Map of Egypt, indicating the geographic position of Moghra. In the inset, map of Africa, indicating the location of Egypt. Map modified from Wikimedia (2017, CC BY SA 3.0: https://commons.wikimedia.org/wiki/File:EGY_orthographic.svg).

The Moghra area (also known in the literature as Moghara or Wadi Moghara) is located at in the northern Western Desert, Matruh Governorate, Egypt, around 60 km south of El Alamein (N30°10′ to 30°30 and E28°30′ to 29°E; Fig. 1). The exposed Miocene Moghra Formation is comprised of about 400 m of siliclastic sediments (Abdel Gawad, 2011; Abdel Gawad, 2016; Hassan, 2013; Abdel Gawad et al., 2012; Abdel Gawad et al., 2014; Abdel Gawad et al., 2016; Hassan et al., 2012).

**Figure 2:**
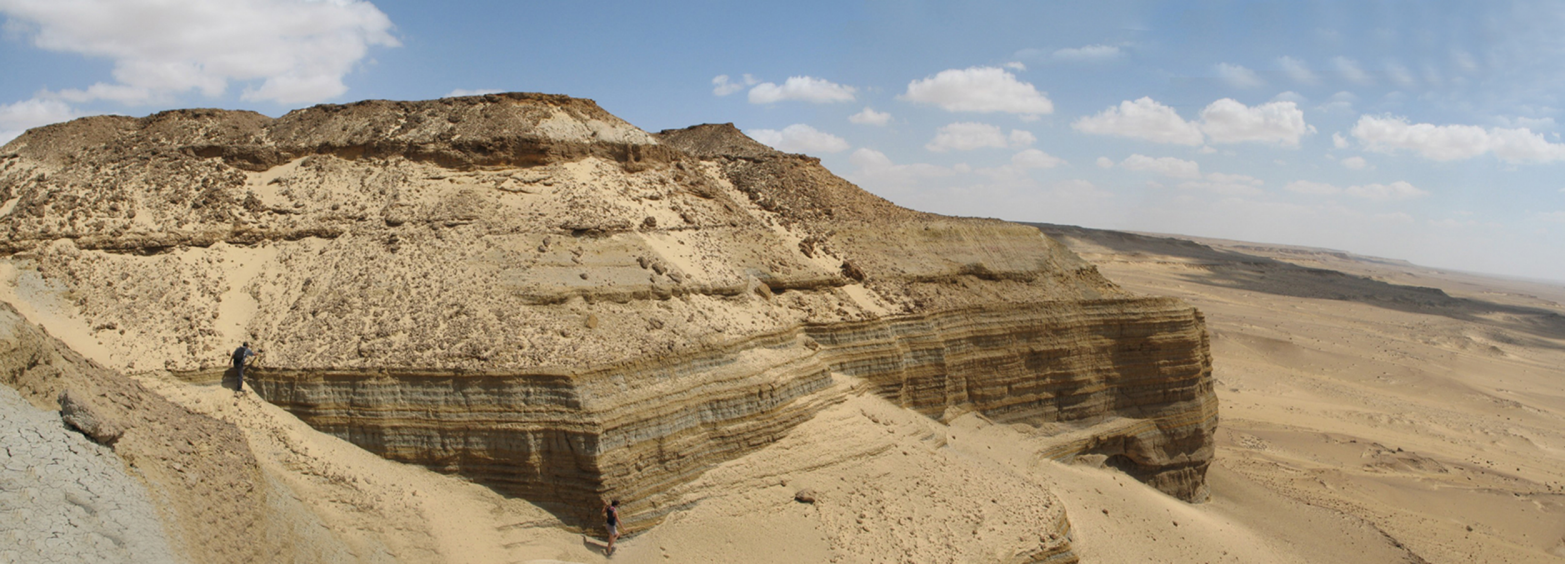
Panoramic photograph of the Moghra Formation. Photograph by ANE­B.

The early Miocene sediments of the Moghra Formation are well exposed in the northeastern escarpment of the Qattara Depression and low hills within the depression, close to the Moghra Oasis, where they dip northward at no more than a few degrees (Fig. 2). To the north, the early Miocene sediments are overlain by the escarpment-capping middle Miocene limestone (Marmarica Formation; Rizk & Davis, 1991; Hassan et al., 2012). The Moghra Formation was named by Said (1962) but has subsequently been interpreted only in very general terms, for example as fluviomarine, semicontinental, and estuarine sediments (Abdallah, 1966), or as fluviomarine sediments (Marzouk, 1970), shallow marine to neritic and as restricted mixed fluviomarine (Khaleifa & Abu Zeid, 1985). Hassan et al. (2012) described the Moghra Formation as a sandy estuarine complex consisting of a series of stratigraphic units that reflect repeated transgressive to regressive shoreline movements across the Burdigalian (early Miocene) coastal landscape. Hassan et al. (2012) identified nine transgressive–regressive units of the Moghra Formation; each of these units is capped by a river-scour surface that severely truncates the underlying regressive half-unit. The transgressive part of each unit is comprised of tidal-fluvial sandstones, within tree trunks and vertebrate bones with cross-stratified, tidal estuarine channel deposits, and ending with open-marine, shelf mudstones, and limestones (Hassan et al., 2012). In short, the palaeonvironment of Moghra has been suggested to be a series of estuarine units stacked in a net transgressive stratigraphy (tide dominated estuary environment) (Hassan, 2013). Already by the early 20th century, Fourtau (1918) proposed two probable depositional environments for the Moghra clastics: a fluviatile terrestrial origin for the lower horizons, as evidenced by the presence of vertebrate fauna; and a marine origin for the upper horizons which contain abundant marine invertebrates.

As a matter of fact, the lower units contain the main four vertebrate fossil-bearing horizons (Abdel Gawad et al., 2010). The existing vertebrate fauna has been reported in four specific stratigraphic horizons found on the basal part of units II, VI, VIII, and X, and the fossil horizons are known as F1, F2, F3, and F4, respectively (Abdel Gawad et al., 2010; Abdel Gawad et al., 2012; Abdel Gawad, 2011). Each horizon represents an erosional lag surface composed of mudclasts associated with coprolites and silicified wood.

^87^Sr/^86^Sr dating showed that the Moghra sequence ranges in age from 21 Ma near the base of the section to around 17 Ma at its top. It has to be highlighted that the Moghra fossil assemblage represents a time-averaged sample. Most of the fossils (including all specimens from the CUWM collection) are derived from the lower–middle part of the section (fossil horizon F1), dated between 19.6–18.2 Ma, however, a few specimens are derived from deposits approaching 17 Ma (Hassan, 2013; see also Morlo et al., 2019).

The Moghra fauna preserves a high diversity and abundance of early Miocene mammals, reptiles, birds, and fishes. It is characterized by nice preservation of the fossils in the sediments. Fossil vertebrates from Moghra are well known since the late 19th century (Jennings-Bramley, 1897; Andrews, 1899; Andrews, 1900; Blanckenhorn, 1901; Fourtau, 1918; Fourtau, 1920). The locality is nevertheless primarily known and mostly famous for its diverse mammal fauna (Rasmussen, Tilden & Simons, 1989; Miller & Simons, 1998; Miller, 1999; Sanders & Miller, 2002; Pickford, Miller & El-Barkooky, 2010; Miller et al., 2014; Morlo et al., 2019). Bird (Miller, Rasmussen & Simons, 1997; Smith, 2013) and fish (Cook et al., 2010; Abdel Gawad et al., 2016) remains are also known from Moghra. As for reptiles, turtle remains from Moghra are the most abundant and they are already known since the very end of the 19th century (Andrews, 1900; Reinach, 1903; Dacqué, 1912; Fourtau, 1920; Gaffney et al., 2011; Abdel Gawad et al., 2014; Abdel Gawad et al., 2018; Abdel Gawad, 2016). These comprise *Trionyx senckenbergianus* Reinach, 1903 (now considered to be a nomen dubium, representing an indeterminate pan-trionychid (see Georgalis & Joyce, 2017)) and a large number of pleurodires: *Mogharemys blanckenhorni* (Dacqué, 1912), *Lemurchelys diasphax* Gaffney et al., 2011, and perhaps also *Latentemys plowdeni* Gaffney et al., 2011, while the two taxa “*Podocnemis*” *aegyptiaca* Andrews, 1900, and “*Podocnemis*” *bramlyi* Fourtau, 1920 (currently considered as nomina dubia by Gaffney et al. (2011) also originate from Moghra. Crocodylian remains have also been preliminarily mentioned (Andrews, 1900; Blanckenhorn, 1901; Abdel Gawad et al., 2014; Abdel Gawad, 2016), which nevertheless reveal the presence of four different lineages (genera *Crocodylus*, *Euthecodon*, *Rimasuchus*, and *Tomistoma*; Abdel Gawad et al., 2014; Abdel Gawad, 2016).

## Material and Methods

All studied fossil material described herein is permanently curated at CUWM and DPC. The material was collected during field work at Moghra, which was approved by the Government of Egypt. Comparative skeletal material of extant varanids and pythonids was studied at the collections of HNHM, MDHC, MNCN, NHMW, and ZZSiD. Comparative fossil material of extinct lizards and snakes was studied in GMH, MNHN, NHMUK, NHMW, PIMUZ, and UU.

## Systematic Palaeontology

**Table:** 

**Figure 3:**
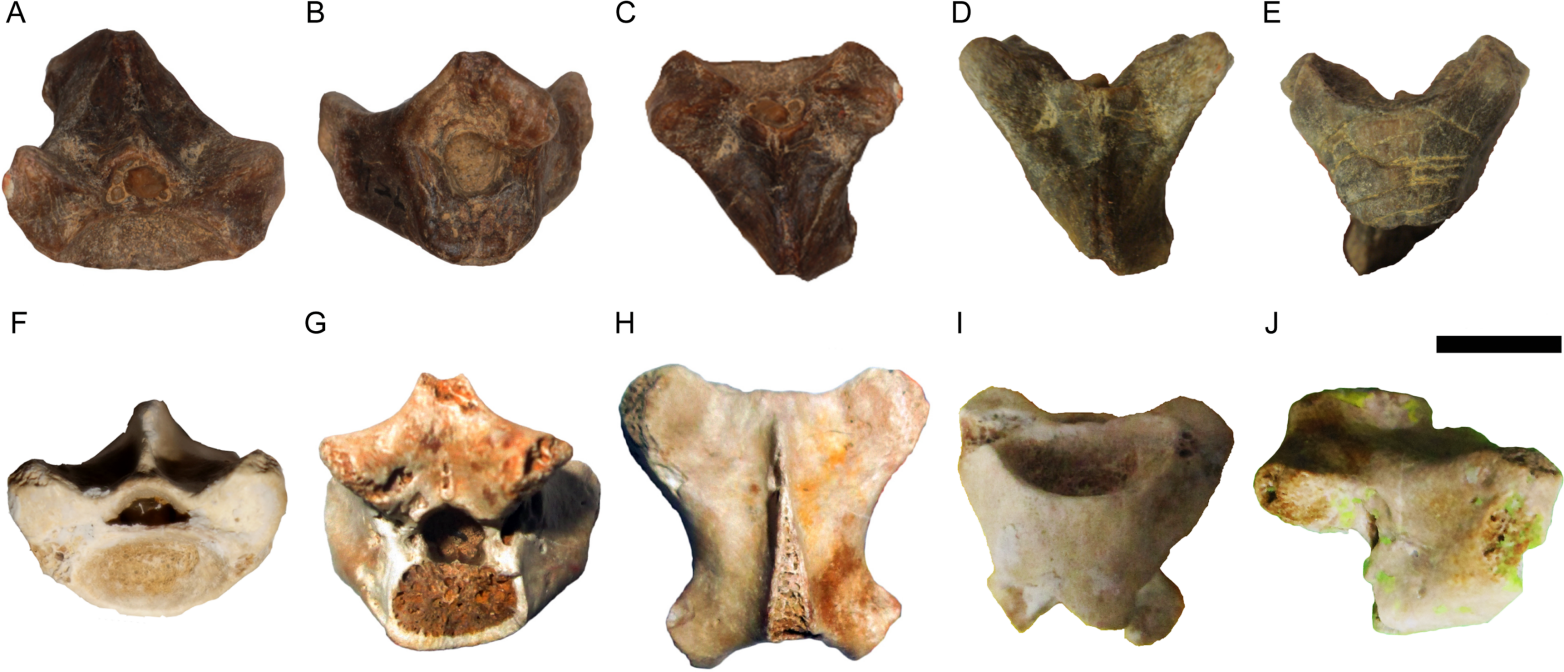
*Varanus.* sp. from Moghra. (A–E) Presacral vertebra DPC 7511 in anterior (A), posterior (B), anterodorsal (C), dorsal (D), and posteroventral (E) views; (F–J) presacral vertebra CUWM 147 in anterior (F), posterior (G), dorsal (H), ventral (I), and right lateral (J) views. Scale bar = 10 mm. Photographs by Ellen Miller and MAG.

REPTILIA Laurenti, 1768
SQUAMATA Oppel, 1811
ANGUIMORPHA Fürbringer, 1900
VARANIDAE Gray, 1827 (sensu Estes, De Queiroz & Gauthier, 1988)
Genus *VARANUS*Merrem, 1820
*Varanus* sp.
(Fig. 3)

**Material**—Two presacral vertebrae (CUWM 147; DPC 7511).

**Description**—Both vertebrae are large and incomplete (Fig. 3). DPC 7511 lacks the condyle, part of the neural spine, and left postzygapophysis, while CUWM 147 lacks the condyle and part of the neural spine. Both vertebrae are procoelous. The cotyle is strongly elliptical and dorsoventrally depressed. As is common for *Varanus*, the cotyle faces anteroventrally so that, in ventral view, the inner surface of the cotyle is largely visible. The prezygapophyses are much dorsally tilted in anterior view, while in dorsal view, they markedly extend anterolaterally. The prezygapophyseal articular facets are large and oval in CUWM 147, while they are distinctly elongated in DPC 7511. A distinct “pars tectiformis” (sensu Hoffstetter, 1969) is present in the anterior part of the neural arch. In posterior view, the neural arch is relatively vaulted. The postzygapophyses are more complete in CUWM 147 and, in dorsal view, they extend posterolaterally. No “pseudozygosphene” or “pseudozygantrum” (sensu Hoffstetter, 1969) is present. Fibrous striae are present in the neural arch of both vertebrae.

**Remarks**—The two presacral vertebrae can be referred to *Varanus* on the basis of the markedly depressed dorsoventrally cotyle and condyle, the cotyle facing anteroventrally, the anteriorly inclined neural arch with a distinct anterior part (“pars tectiformis”), and the presence of striae on the neural arch (Bailon & Rage, 1994; Smith, Bhullar & Holroyd, 2008; Delfino et al., 2013; Georgalis et al., 2018; Ivanov et al., 2018; Villa et al., 2018). The occurrence of a marked precondylar constriction, a diagnostic feature of *Varanus* (Estes, 1983; Smith, Bhullar & Holroyd, 2008), cannot be evaluated. The Moghra vertebrae bear some overall resemblance with those of *Varanus rusingensis* from the early Miocene of Kenya, which is so far the only named fossil varanid from Africa (Clos, 1995). However, vertebrae of *Varanus* are variable and do not possess diagnostic features for species distinguishment (see Georgalis et al., 2018). Accordingly, and taking into consideration also the fragmentary nature of the Egyptian material, we attribute it only to the genus level.

**Table:** 

**Figure 4:**
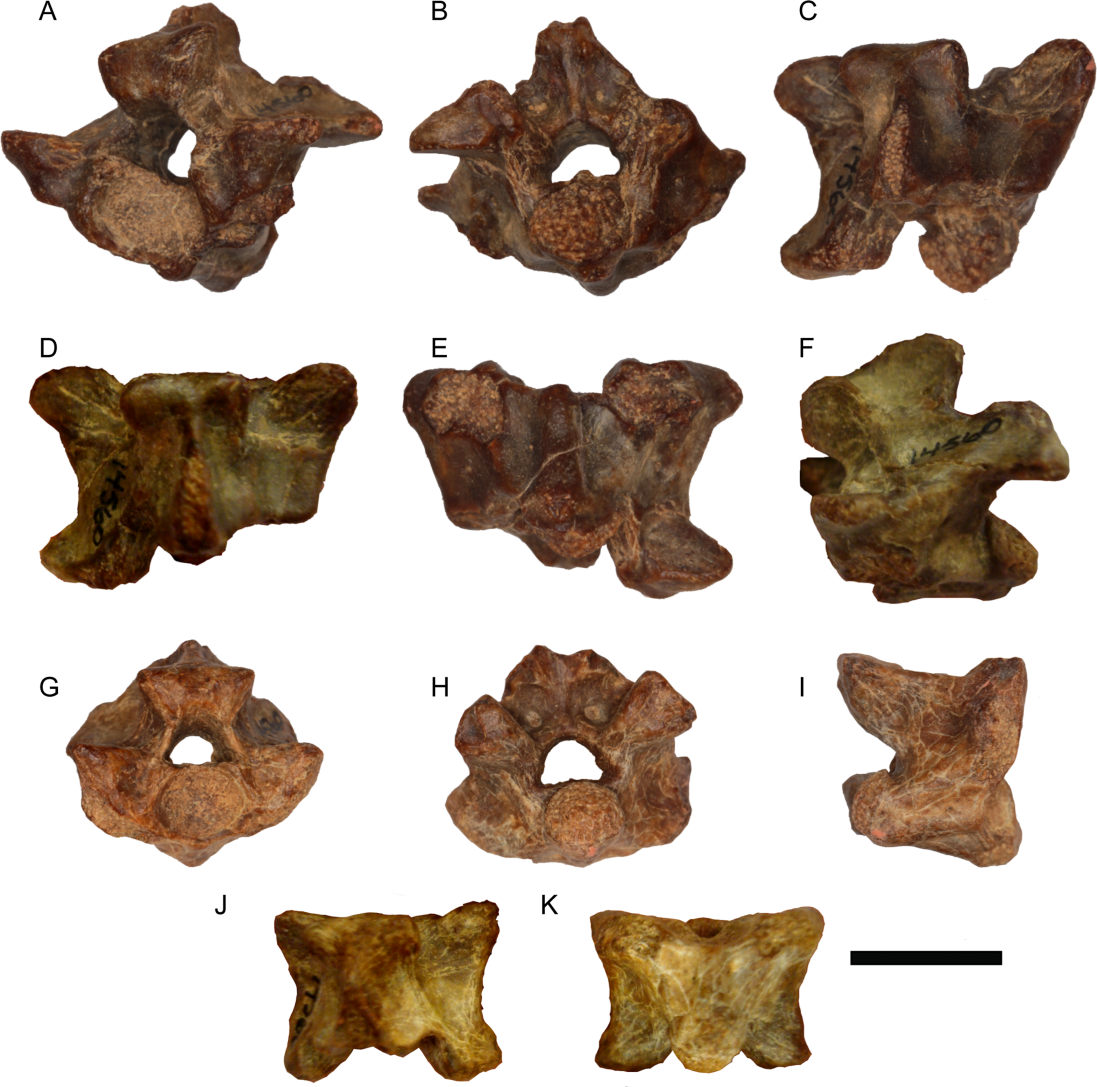
*Python.* sp. from Moghra. (A–F) Trunk vertebra DPC 14600 in anterolateral (A), posterior (B), dorsolateral (C), dorsal (D), ventral (E), and left lateral (F) views; (G–K) trunk vertebra DPC 14560 in anterior (G), posterior (H), right lateral (I), dorsal (J), ventral (K), and views. Scale bar = 10 mm. Photographs by Ellen Miller.

**Figure 5:**
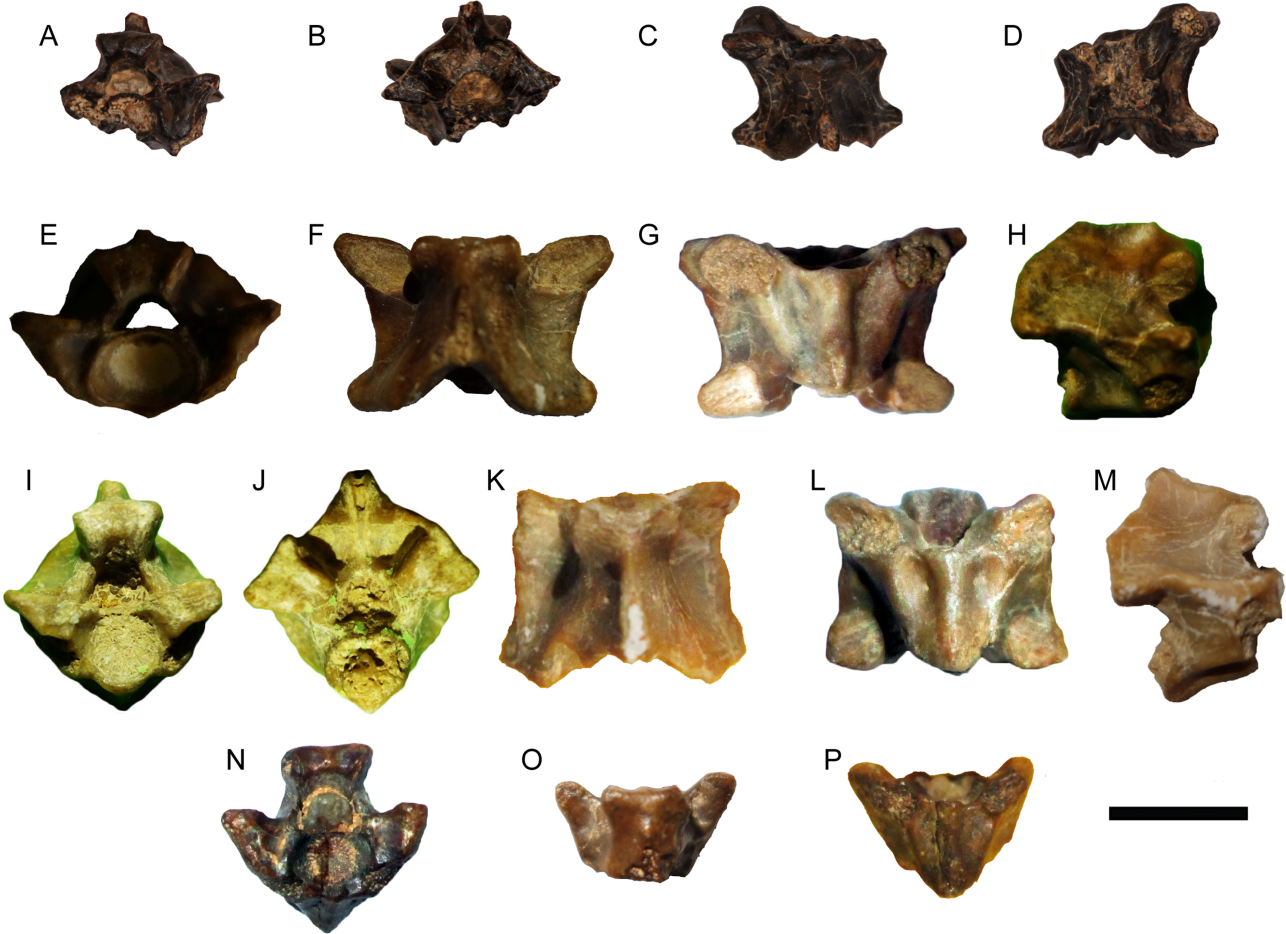
*Python.* sp. from Moghra. (A–D) Trunk vertebra DPC 14530 in anterior (A), posterior (B), dorsal (C), and ventral (D) views; (E–H) trunk vertebra CUWM 137 in anterior (E), dorsal (F), ventral (G), and right lateral (H) views; (I–M) trunk vertebra CUWM 160 in anterior (I), posterior (J), dorsal (K), ventral (L), and right lateral (M) views; (N–P) trunk vertebra CUWM 9 in anterior (N), dorsal (O), and ventral (P) views. Scale bar = 10 mm. Photographs by Ellen Miller and MAG.

SERPENTES Linnaeus, 1758
PYTHONIDAE Fitzinger, 1826
Genus *PYTHON*Daudin, 1803
*Python* sp.
(Figs. 4 and 5)

**Material**—Six trunk vertebrae (CUWM 9; CUWM 137; CUWM 160; DPC 14530; DPC 14560; DPC 14600).

**Description**—The vertebrae are all moderately large with centrum lengths ranging between 6 and 11 mm. CUWM 137 (centrum length = 8.8 mm) is nearly complete, though still lacks most of the neural spine. CUWM 160 (centrum length = 8.0 mm) lacks the dorsal part of the neural spine and part of the left prezygapophysis, while its paradiapophyses, cotyle, and condyle are eroded. DPC 14560 (centrum length = 8.9 mm), lacks part of the neural spine and the posteriormost portion of the neural arch. DPC 14600 is the largest vertebra (centrum length = 10.9 mm) and lacks its right postzygapophysis and the dorsal portion of the neural spine. DPC 14530 (estimated centrum length around 7 mm) lacks much of the cotyle, right prezygapophysis, neural spine, and the anteroventral and posteroventral portions of the centrum. CUWM 9 (centrum length = 6.1 mm) lacks most of the posterior portion of the neural arch, neural spine, and both postzygapophyses, while its condyle and paradiapophyses are strongly eroded. In all vertebrae, the centrum is distinctly wider than long. The zygosphene is thick, massive, and with a zygosphenal tuberosity (sensu Head, 2005) in anterior view. The thickness of the zygosphene is most apparent in the largest vertebrae (e.g., DPC 14560 and DPC 14600), whereas in CUWM 160 and especially CUWM 9, the zygosphene is thinner than in the other specimens. In dorsal view, the zygosphene possesses three more or less well-delimited lobes, though exceptions where the lobes are almost incipient still exist (e.g., DPC 14600). The prezygapophyses are slightly dorsally tilted. The prezygapophyseal articular facets are large and relatively elongated. There are no paracotylar foramina. The cotyle is large and slightly dorsoventrally depressed. The condyle is large and relatively circular. The neural arch is vaulted and is distinctly upswept above the zygantrum. A distinct angle is present at around the mid-length of each postzygapophysis in posterior view. The posterior median notch of the neural arch is deep. The interzygapophyseal constriction is shallow. In ventral view, the centrum is widened anteriorly. The width of the haemal keel varies, depending on the position of the vertebra in the column, with the posterior trunk vertebrae possessing an even wider haemal keel (e.g., CUWM 137) in comparison with mid-trunk ones (e.g., CUWM 9; CUWM 160). The subcentral grooves are deep, being even deeper in posterior trunk vertebrae. The paradiapophyses are massive and are not clearly divided into diapophyseal and parapophyseal portions. The postzygapophyseal articular facets are massive and oval. The zygantrum is massive and deep. The neural spine is damaged in most specimens. When preserved, it appears that the neural spine begins to grow gradually in height towards the posterior portion of the neural arch (e.g., CUWM 160; DPC 14600). The full height of the neural spine cannot be evaluated with certainty, though from CUWM 160 and DPC 14600 it can be tentatively inferred that this structure was not very high. In dorsal view, the neural spine commences well behind the zygosphene.

**Remarks**—The vertebrae can be referred to the genus *Python* on the basis of their overall large size and massive construction, centrum length wider than long, massive and thick zygosphene with tuberosity in anterior view, shallow interzygapophyseal constriction, massive paradiapophyses not divided into diapophyseal and parapophyseal portions, and absence of paracotylar foramina (characters from Rage, 1984; Ivanov, 2000; Szyndlar & Rage, 2003; Head, 2005). Differences in the overall vertebral size, the thickness and width of the zygosphene, the vaulting of the neural arch, and the width of the haemal keel, can be attributed to intracolumnar variation and as such, they do not suggest the distinction of different *Python* species in the assemblage. It seems that the Moghra python was characterized by a relatively low neural spine, though this assumption cannot be verified with certainty as this element is not fully preserved in any of the available fossil vertebrae. If this assumption is correct, then in this respect, the Moghra material approaches the condition of *Python euboicus* Römer, 1870, from the early Miocene of Greece and especially *Python europaeus* Szyndlar & Rage, 2003, from the early and middle Miocene of Central and Western Europe (see figures in Römer, 1870 and Szyndlar & Rage, 2003). The Moghra material can be differentiated from the sole so far named extinct pythonid from Africa, i.e., *Python maurus*, from the middle Miocene of Morocco (Rage, 1976), by its more depressed neural arch and (perhaps also) lower neural spine. Nevertheless, the Egyptian and Moroccan forms share in common a number of characters, such as the rather thick and triangular zygosphene, the deep zygantrum, and the shape and size of the postzygapophyseal articular facets. Pending the discovery of additional and more complete pythonid material from Moghra, we herein refrain from suggesting any potential close or conspecific affinities with either the European (*Python euboicus* and *P. europaeus*) or the African species (*P. maurus*).

## Discussion

**Figure 6:**
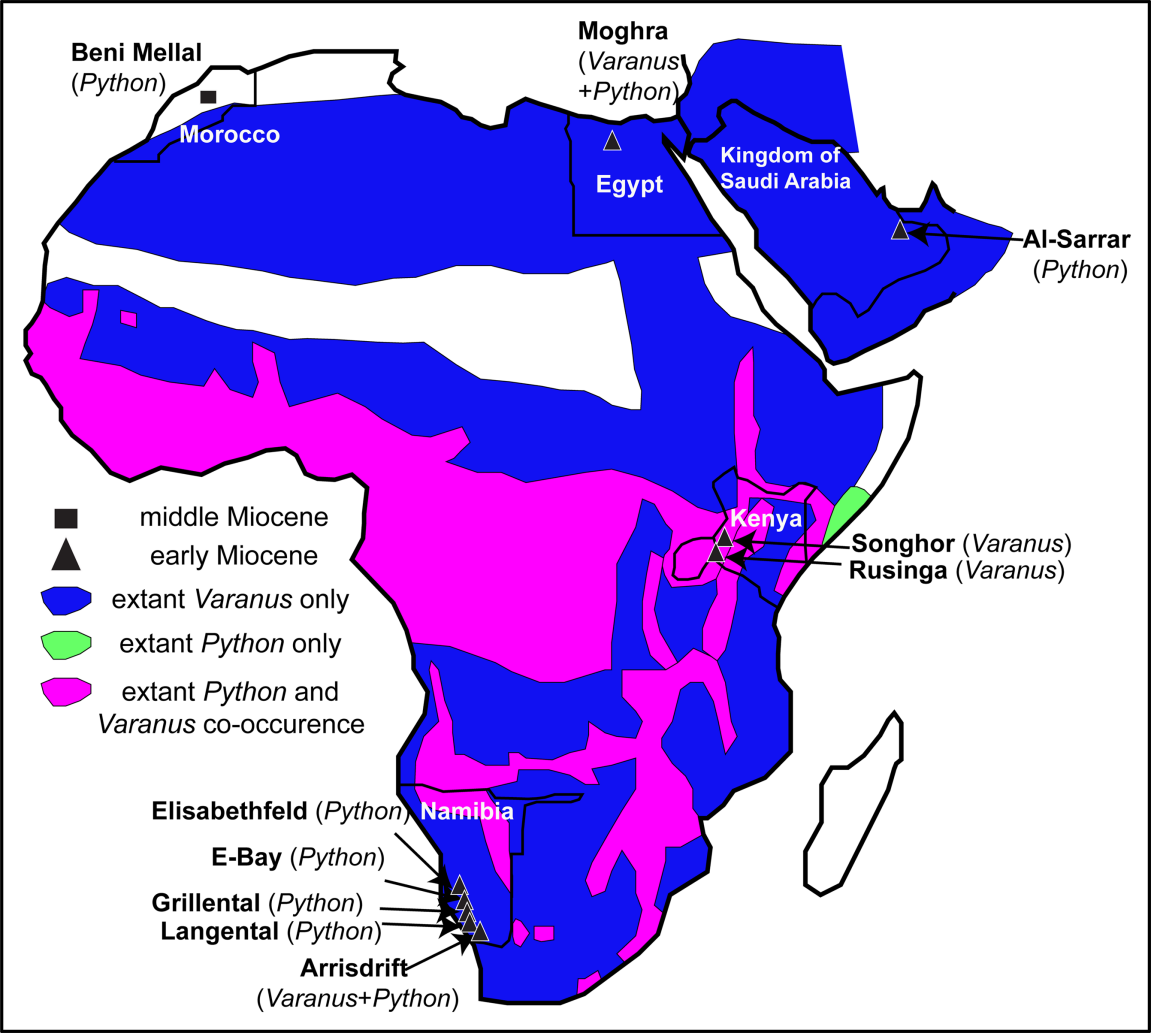
Map of Afro-Arabia indicating all early and middle Miocene localities from where fossil remains of *Varanus* and or *Python* have so far been descri bed. The map also indicates the ranges of extant *Varanus* (blue colour) and *Python* (green colour) in Afro-Arabia, and the areas where both genera co-occur (purple colour). Ranges of extant taxa follow Pianka, King & King (2004) for *Varanus* and Wallach, Williams & Boundy (2014) for *Python*. Scale bar = 800 km. Map modified from Wikimedia (2019, CC BY SA 3.0: https://commons.wikimedia.org/wiki/File:African_Union_(orthographic_projection).svg?wprov=srpw1_0).

The largest and perhaps among the most iconic squamates from Africa, *Varanus* and *Python*, occur sympatrically throughout much of the continent and the fossil record demonstrates that this sympatry may have occurred much earlier (Fig. 6). Unfortunately, the patchiness of the squamate fossil record from Africa cannot afford any precise patterns or the full extent of this ecological sympatry through time.

Varanids are known in the African fossil record since the late Eocene. Indeed, *Varanus* or a *Varanus*-like form probably pertaining to the stem of this lineage is already present in Egypt since the late Eocene (early Priabonian) (Holmes et al., 2010a). Additional, probably congeneric remains as well as indeterminate varanids also exist from the early Oligocene of Egypt (Smith, Bhullar & Holroyd, 2008; Holmes et al., 2010a). The earliest Neogene record in Africa that can be securely assigned to the genus *Varanus* originates from much younger strata, being represented by *Varanus rusingensis* from the early Miocene (Burdigalian) of Rusinga Island, Kenya (Clos, 1995), another (conspecific or closely related) form from the early Miocene of Songhor, Kenya (Clos, 1995), and at least one indeterminate form from the early Miocene (Burdigalian) of Arrisdrift, Namibia (Rage, 2003). No other Miocene material of *Varanus* has been formally described from Africa. Additionally, the genus is scarcely known in Plio-Pleistocene localities across Africa (Delfino et al., 2004; Delfino et al., 2018; Head & Müller, 2020).

*Python* is not well documented in the African fossil record. Paleogene records of large “booids” in Africa do exist (e.g., McCartney & Seiffert, 2016), however, they pertain to totally different lineages than the extant *Python*, some bearing potential affinities with the Eocene European genus *Palaeopython* Rochebrune, 1880. As far as it regards strictly *Python*, the earliest record of the genus in the continent originates from the early Miocene localities Grillental, Elisabethfeld, E-Bay, Arrisdrift, and Langental, all of them in Namibia (Rage, 2003; Rage, 2008). The Namibian remains have been tentatively referred only to the genus level (Rage, 2008), with the exception of the one from the locality of Arrisdrift that has been tentatively assigned to *Python sebae* (Gmelin, 1789), an extant species that is widespread in sub-Saharan Africa (*Python* cf. *P. sebae* of Rage, 2003). Nevertheless, that Arrisdrift *Python* material has never been figured. Moghra is at least slightly older than Arrisdrift, though generally it is considered slightly younger than Grillental, Langental, and Elisabethfeld (e.g., Morales, Pickford & Valenciano, 2016). However, while the Moghra Formation spans a time range within the Burdigalian, most of the new Egyptian specimens (including all from the CUWM collection) have an age between 19.6 and 18.2 Ma; thus they could be indeed even older than all Namibian congeners. In any case, the new *Python* remains rank as among the oldest of the genus from Africa. Other Miocene occurrences of the genus in Africa also exist sporadically across the continent (Rage, 1976; Bailon & Rage, 1994), including *Python maurus*, known exclusively from its type locality, the middle Miocene (MN 6) of Beni Mellal, Morocco, which represents the sole named extinct pythonid taxon from Africa (Rage, 1976). Material from the late Miocene of Sahabi, Libya, described by Hecht (1987) could also pertain to *Python*. Outside Africa (sensu stricto), *Python* has been reported also from the early Miocene of Saudi Arabia (Rage, 1982), with that area being part of the Afro-Arabian plate during that time anyway. *Python* is also known from Pliocene and Quaternary sediments in Africa (Rage, 1973; Meylan, 1987; Delfino et al., 2004; Delfino et al., 2018; Head & Müller, 2020).

Among these early Miocene Namibian and Kenyan localities that yielded remains of *Varanus* or *Python*, only in Arrisdrift have fossils of both genera been recovered (Rage, 2003), as all the remaining ones yielded either *Varanus* or *Python* fossils only. Therefore, the new remains from Moghra (at least the specimens from the CUWM collection, all originating from the lower–middle part of the section [F1; 19.6–18.2 Ma]) mark the oldest co-occurrence of the genera *Varanus* and *Python* in Africa and document that this close sympatry between these two large squamates occurred in Africa already by the early Miocene (Burdigalian) and outside the modern geographic extent of their co-distribution (Fig. 6).

Interestingly, the Burdigalian coincides with the first appearance of these two genera also in the fossil record of Europe. Indeed, the oldest *Varanus* specimens from Europe are also known from that time (Hoffstetter, 1969; Augé & Guével, 2018; Rage & Bailon, 2005; Delfino et al., 2013; Ivanov et al., 2018; see Table 1). These early Miocene remains of *Varanus* from Europe appear almost simultaneously across different areas of the continent (Czech Republic, France, Spain), denoting a rapid geographic expansion of the genus during that time (Table 1). No early Miocene varanid remains are known from the Balkans or Anatolia, but it has to be underlined that both these areas have yielded so far only rather few reptilian finds of that age (see Georgalis et al., 2019). *Varanus* so far first appears in the Balkans only as early as the middle Miocene (MN 6) of Subpiatră 2/1, Romania (Venczel et al., 2005; Hír & Venczel, 2005) and Prebreza, Serbia (Milosević, 1967), with the latter originally identified as a tortoise (Milosević, 1967; though the same slab consists of both testudinid and varanid remains). Nevertheless, *Varanus* is no longer present in the extant herpetofauna of the continent although it existed there until relatively recently, with its last occurrence being documented from the Middle Pleistocene of Greece (Georgalis, Villa & Delfino, 2017). On the other hand, in Europe, the fossil record of *Python* is more adequately known in comparison with the African one, though still the earliest occurrences of the genus are again of early Miocene (Burdigalian) age, with the genus becoming ultimately extinct in the continent shortly thereafter, during the middle Miocene (Römer, 1870; Ivanov, 2000; Szyndlar & Rage, 2003; Rage & Bailon, 2005; Ivanov & Böhme, 2011).

**Table 1:** List of all known early and middle Miocene localities from Africa, Europe, and Asia, from where fossil remains of *Varanus* and / or *Python* have so far been formally described.

**Locality**	**Taxa**	**References**
Kymi, Euboea Island, Greece; early Miocene, MN 3/4	*Python euboicus* (TL)	Römer (1870)
Mokrá-Western Quarry, Moravia, Czech Republic; early Miocene, MN 4	*Varanus mokrensis* (TL)	Ivanov et al. (2018)
Moghra, Egypt; early Miocene, Burdigalian	*Varanus* sp.; *Python* sp.	This paper
Elisabethfeld, Namibia; early Miocene, Burdigalian	*Python* sp.	Rage (2008)
Songhor, Kenya; early Miocene, Burdigalian	?*Varanus rusingensis*	Clos (1995)
Grillental, Namibia; early Miocene, Burdigalian	cf. *Python* sp. A	Rage (2008)
Langental, Namibia; early Miocene, Burdigalian	cf. *Python* sp. B	Rage (2008)
Rusinga Island, Kenya; early Miocene, Burdigalian	*Varanus rusingensis* (TL)	Clos (1995)
E-Bay, Namibia; early Miocene, Burdigalian	?cf. *Python* sp. A	Rage (2008)
Arrisdrift, Namibia; early Miocene, Burdigalian	*Varanus* sp.; *Python* cf. *sebae*	Rage (2003)
Artenay, Loiret, Centre-Val de Loire, France; early Miocene, MN 4	*Varanus* sp.	Hoffstetter (1969) and Augé & Guével (2018)
Béon 1 (= Montréal-du-Gers), Gers, France; early Miocene, MN 4	*Varanus* sp.; *Python europaeus*	Rage & Bailon (2005)
Al-Sarrah, Eastern Province, Saudi Arabia; early Miocene, MN 4	*Python* sp.	Rage (1982)
Can Mas, Vallés-Penedés, Catalonia, Spain; early Miocene, MN 4	*Varanus* sp.	Hoffstetter (1969) and Delfino et al. (2013) (type of *Iberovaranus catalaunicus*)
Ayakoz, Eastern Kazakhstan, Kazakhstan; early Miocene	*Varanus* sp.	Malakhov (2005)
Etadunna Formation, Australia; early Miocene	?*Varanus* sp.	Estes (1984)
Hiatus and White Hunter localities, Riversleigh, Australia; early Miocene	*Varanus* sp.	Scanlon (2014)
Vieux-Collonges (= Mont Ceindre), Auvergne-Rhône-Alpes; early-middle Miocene, MN 4/5	*Varanus* cf. *hofmanni*; *Python europaeus* (TL)	Hoffstetter (1969), Ivanov (2000)) and Szyndlar & Rage (2003)
La Grive ”old levels” (Fissure P&B), France; middle Miocene, MN 5	*Python europaeus*	Szyndlar & Rage (2003)
Pontigné, Maine-et-Loire, Pays de la Loire, France; middle Miocene, MN 5	*Varanus* sp.	Gobé, Mornand & Pouit (1980)
Amor, Leiria, Portugal; middle Miocene, MN 5	*Varanus* sp.	Antunes & Mein (1981)
Siwaliks, localities “Y-802; Y-642; Y-478; Y-650; Y-882”, Potwar Plateau, Pakistan; middle Miocene, 16.8–13 Ma	*Python* sp.	Head (2005)
Griesbeckerzell 1a + 1b, Bavaria, Germany; middle Miocene, MN 5/6	*Python* sp.	Ivanov & Böhme (2011)
Beni Mellal, Tadla-Azilal, Morocco; middle Miocene, MN 6	*Python maurus* (TL)	Rage (1976)
Prebreza, Serbia; middle Miocene, MN 6	*Varanus* sp.	Milosević (1967)
Litke 2, Hungary; middle Miocene, MN 6	*Varanus* sp.	Venczel & Hír (2015)
Subpiatră 2/1, Bihor, Romania; middle Miocene, MN 6	*Varanus* sp.	Venczel et al. (2005) and Hír & Venczel (2005)
Bujor, Moldova; middle Miocene, MN 7/8	*Varanus lungui* (TL)	Lungu, Zerova & Chkhikvadze (1983) and Zerova & Chkhikvadze (1986)
Varnitza (= Varnitsa), Moldova; middle Miocene, MN 7/8	*Varanus tyrasiensis* (TL)	Lungu, Zerova & Chkhikvadze (1983) and Zerova & Chkhikvadze (1986)
Gratkorn, Styria, Austria; middle Miocene, MN 7/8	*Varanus* sp.	Böhme & Vasilyan (2014)
La Grive (= La Grive-Saint-Alban), Isère, Auvergne-Rhône-Alpes, France; middle Miocene, MN 7/8	*Varanus* cf. *hofmanni*	Fejérváry (1918) and Hoffstetter (1969)
Abocador de Can Mata, Catalonia, Spain; middle Miocene, MN 7/8	*Varanus marathonensis*	Villa et al. (2018)
Mynsualmas, Western Kazakhstan, Kazakhstan; middle Miocene	*Varanus pronini* (TL)	Zerova & Chkhikvadze (1986)
Mochiwala, Chinji Formation, Pakistan; ?middle Miocene	*Python* sp.	Hoffstetter (1964)

**Notes.**

TL type locality

Furthermore, the Asian fossil record of both *Varanus* and *Python* is rather scarce, being mostly confined to late Neogene and Quaternary occurrences (e.g., Lydekker, 1888; Hoffstetter, 1964; Rage, Gupta & Prasad, 2001; Head, 2005; Suraprasit et al., 2016). The earliest confirmed Asian record of *Varanus* originates from the early Miocene of Kazakhstan (Malakhov, 2005). Supposed records of the same genus from the middle Eocene and early Oligocene of Mongolia have been reported by Alifanov (1993) and Alifanov (2012), however, these have either briefly described and/or figured or either simply mentioned; their attribution to *Varanus* has been disputed (e.g., Rage & Bailon, 2005). We consider that the Mongolian records most likely pertain to some non-*Varanus* genus, such as *Saniwa* Leidy, 1870, which was after all present in the Paleogene of Asia (Pianka, King & King, 2004). The earliest Asian record of *Python* is from the middle Miocene of Pakistan (Head, 2005), being thus certainly younger from both African and European congeneric forms. In Australia, *Varanus* is known from several late Neogene and Quaternary localities (Owen, 1859; Pianka, King & King, 2004; Hocknull et al., 2009); the earliest occurrences of the genus there seem to have been around the early Miocene (Table 1), however, none of these have been adequately described or figured (see Scanlon, 2014). Pythonids are widespread and diverse in Australia but all pertain to genera other than *Python* (Wallach, Williams & Boundy, 2014).

In fact, the Burdigalian was a key time interval for major dispersal events of terrestrial vertebrate groups between Africa and Europe as it coincides with the collision of the Afro-Arabian plate with Eurasia (Rögl, 1999). Such collision resulted in the formation of the so called “*Gomphotherium* Landbridge”, which enabled direct dispersal of numerous terrestrial vertebrate groups (Sanders & Miller, 2002; Koufos, Zouros & Mourouzidou, 2003), including also multiple reptile lineages (Čerňanský, 2012; Georgalis, Villa & Delfino, 2016; Čerňanský et al., 2017; Čerňanský & Syromyatnikova, 2019; Georgalis et al., 2019). In particular, the Levant has played an active role to such early Miocene dispersal events between Eurasia and Africa (“Levantine corridor”; López-Antoñanzas et al., 2016; Grossman et al., 2019), while recent evidence witnesses that this pivotal biogeographic role continued also during the late Miocene (López-Antoñanzas et al., 2019). This has been particularly documented for mammals, such as anthracotheriids and rodents (López-Antoñanzas et al., 2016; López-Antoñanzas et al., 2019; Grossman et al., 2019).

**Figure 7:**
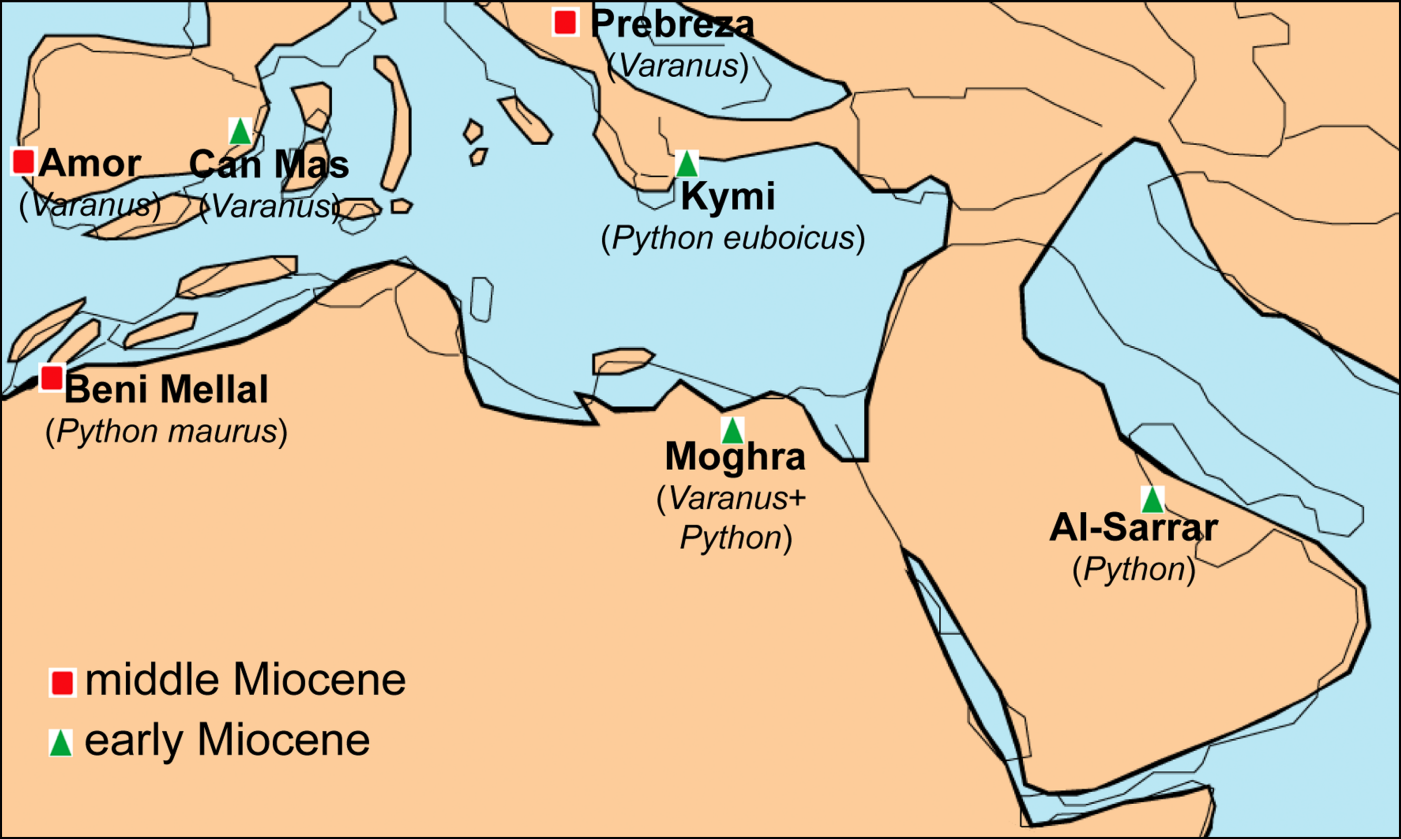
Palaeogeographic reconstruction of the Mediterranean region during the late early to early middle Miocene, showing all the localities that have yielded fossil remains of *Varanus* and *Python*. Map modified from Rögl (1999), with data also from Popov et al. (2004).

The geographic location of Moghra, being situated rather close to the “*Gomphotherium*-Landbridge” and particularly to the “Levantine corridor” offers new insight into the biogeography of various vertebrate groups. Indeed, certain mammals from the Moghra fauna have been suggested to represent immigrants from Eurasia that dispersed to Africa during the early Neogene (e.g., the anthracotheriids; Miller et al., 2014). The identification of *Varanus* and *Python* in the faunal assemblage of Moghra denotes that these large squamates probably also used the “*Gomphotherium*-Landbridge” for their dispersal between Afro-Arabia and Eurasia. Nevertheless, the exact route of dispersal is still obscure. The exact origins of pythonids are still a matter of debate, with an Eurasian origin sometime during the Paleogene having been tentatively suggested based on morphological data and fossil record (see Scanlon, 2001). Nevertheless, more particularly for the genus *Python*, it has been recently suggested, on the basis of molecular data, that it originated in Africa and from there dispersed to Europe and Asia (Reynolds, Niemiller & Revell, 2014). The current identification of the Moghra *Python* could support this scenario as it is older than the earliest Asian occurrence and almost coeval with the European ones. In addition, one of the earliest European occurrences, i.e., *Python euboicus*, is known from Greece, an area which certain early Miocene African squamates are considered to have used after crossing the “*Gomphotherium-* Landbridge” in order to disperse to the rest of Europe (see Georgalis, Villa & Delfino, 2016; Georgalis et al., 2019)—note that the exact age of Kymi, the type locality of *P. euboicus*, is not well constrained, ranging between MN 3 or MN 4, but in any case it certainly pertains to the Burdigalian (Szyndlar & Rage, 2003; Georgalis et al., 2019). On the other hand, on the basis of molecular data and European fossil specimens, it has been suggested that the lineage of *Varanus* is of Eurasian origin with subsequent dispersal(s) and diversification to Africa (Vidal et al., 2012; Ivanov et al., 2018); however, alternatively Villa et al. (2018) stated that an opposite route (dispersal from Africa to Eurasia during the early Miocene) should not be ruled out, while a Paleogene African origin of *Varanus* is also supported by early Oligocene finds of this genus (or a closely related form) from Fayum, Egypt (Smith, Bhullar & Holroyd, 2008; Holmes et al., 2010a). The fact that early Miocene *Varanus* remains are known from all major continents of the Old Word (Europe, Africa, Asia, and Australia; see Table 1) hampers the understanding of the exact origins and dispersal routes of Neogene monitor lizards. In any case, the identification of both *Varanus* and *Python* from Moghra, renders this Egyptian locality as one of only a few among the whole Mediterranean area that yielded remains of these large squamates (Fig. 7).

The sympatry of *Varanus* and *Python* across the Neogene of Africa, continuing also up to today, is reminiscent of other such cases of large lizards and snakes coexisting together across large geographic distances and stratigraphic spans during the Cenozoic. Such is the case of *Palaeovaranus* and *Palaeopython* found together in multiple localities across the Eocene of Western and Central Europe (see Georgalis & Scheyer, 2019), a case that rather amusingly fits also “euphoniously” to the case of sympatry of *Varanus* and *Python*.

## Conclusions

New varanid and pythonid fossil remains from the early Miocene of Moghra expand the known fossil record of *Varanus* and *Python* in Africa. Furthermore, Moghra marks the earliest co-occurrence of *Varanus* and *Python* in the African fossil record. The sympatric occurrence of *Varanus* and *Python*, a case that is widespread in the extant herpetofauna of Africa, is thoroughly discussed. The geographic location of Moghra, along with its stratigraphic position, seems to have played a pivotal role for dispersal events between African and Eurasian squamates. Furthermore, the identification of a strongly thermophilous reptile, such as *Python*, in the Moghra fossil assemblage provides temperature and climatic constraints and enables a better understanding of the palaeoenvironment of this locality, which is crucial for our understanding of the African Miocene. The new Moghra material adds to the so far poorly known fossil record of squamates from Egypt, so far confined to few but nevertheless rather important descriptions of agamid and varanid lizards and palaeophiid, madtsoiid, “booid”, russellophiid, and colubroid snakes all from the world famous Eocene of Fayum (Andrews, 1901; Andrews, 1906; Janensch, 1906; Smith, Bhullar & Holroyd, 2008; Holmes et al., 2010a; Holmes et al., 2010b; McCartney & Seiffert, 2016; Rio & Mannion, 2017), as well as “colubrine” snakes from the middle Miocene of Khasm El-Raqaba (Gunnell et al., 2016).

## Institutional abbreviations

CUWM: Wadi Moghra collection, Geology Department, Cairo University , Cairo, Egypt
DPC: Duke Lemur Center, Division of Fossil Primates, Duke University, Durham, North Carolina, USA
GMH: Geiseltalmuseum of Martin-Luther Universität Halle-Wittenberg, now referred to as the Geiseltalsammlung, housed as part of the Zentralmagazin Naturwissenschaftlicher Sammlungen, Halle, Germany
HNHM: Hungarian Natural History Museum, Budapest, Hungary
MDHC: Massimo Delfino Herpetological Collection, University of Torino, Italy
MNCN: Museo Nacional de Ciencias Naturales, Madrid, Spain
MNHN: Muséum national d’Histoire naturelle, Paris, France
NHMUK: Natural History Museum, London, United Kingdom
NHMW: Naturhistorisches Museum, Vienna, Austria
PIMUZ: Paläontologisches Institut und Museum der Universität Zürich, Zürich, Switzerland
UU: Department of Geosciences, Utrecht, The Netherlands
ZZSiD: Polish Academy of Sciences, Krakow, Poland

## Additional Information and Declarations

### Field Study Permissions

The following information was supplied relating to field study approvals (i.e., approving body and any reference numbers): Field work at Moghra was approved by the Government of Egypt.

### Data Availability

The following information was supplied regarding data availability: The material described herein is permanently curated at the collections of CUWM (Wadi Moghra collection, Geology Department, Cairo University , Cairo, Egypt) and DPC (Duke Lemur Center, Division of Fossil Primates, Duke University, Durham, North Carolina, USA). Raw data in the Article can be found in the Material and Methods and in the Systematic Palaeontology sections. All specimens: CUWM 147: presacral vertebra of *Varanus* sp.; DPC 7511: presacral vertebra of *Varanus* sp.; CUWM 9: trunk vertebra of *Python* sp.; CUWM 137: trunk vertebra of *Python* sp.; CUWM 160: trunk vertebra of *Python* sp.; DPC 14530: trunk vertebra of *Python* sp.; DPC 14560: trunk vertebra of *Python* sp.; DPC 14600: trunk vertebra of *Python* sp.
